# Self-esteem in patients with inflammatory bowel disease

**DOI:** 10.1007/s11136-020-02467-9

**Published:** 2020-03-06

**Authors:** Randi Opheim, Bjørn Moum, Bjørn Tore Grimstad, Jørgen Jahnsen, Ingrid Prytz Berset, Øistein Hovde, Gert Huppertz-Hauss, Tomm Bernklev, Lars-Petter Jelsness-Jørgensen

**Affiliations:** 1 Department of Gastroenterology, Oslo University Hospital, Oslo, Norway; 2 Department of Nursing Science, Institute of Health and Society, Faculty of Medicine, University of Oslo, Oslo, Norway; 3 Institute of Clinical Medicine, Faculty of Medicine, University of Oslo, Oslo, Norway; 4 Department of Internal Medicine, Stavanger University Hospital, Stavanger, Norway; 5 Department of Clinical Science, University of Bergen, Bergen, Norway; 6 Department of Gastroenterology, Akershus University Hospital, Lørenskog, Norway; 7 Department of Medicine, Aalesund Hospital Trust, Aalesund, Norway; 8 Department of Internal Medicine, Innlandet Hospital Trust, Gjøvik, Norway; 9 Department of Gastroenterology, Telemark Hospital Trust, Skien, Norway; 10 Department of Research and Development, Vestfold Hospital Trust, Tønsberg, Norway; 11 Department of Gastroenterology, Østfold Hospital Trust, Grålum, Norway; 12 Department of Health Sciences, Østfold University College, Fredrikstad, Norway

**Keywords:** Self-esteem, Self-efficacy, Coping, Inflammatory bowel disease, Ulcerative colitis, Crohn’s disease, Health psychology, Chronic disease, Health-related quality of life

## Abstract

**Purpose:**

The purpose of this study was to explore self-esteem and associations between self-esteem and sociodemographic, clinical, and psychological factors in patients with inflammatory bowel disease (IBD)**,** a disease of chronic relapsing inflammation of the gastrointestinal tract. IBD symptoms, including pain, fatigue, and diarrhea, as well as potential life-long medical treatment and surgery, may be demanding, cause significant challenges, and influence self-esteem.

**Methods:**

In this cross-sectional multicenter study, participants were recruited from nine hospitals in the southeastern and western regions of Norway from March 2013 to April 2014. Data were collected using self-report questionnaires. Self-esteem was assessed by the Rosenberg Self-Esteem Scale, fatigue was assessed by the Fatigue Questionnaire, self-efficacy was assessed by the General Self-Efficacy Scale, and disease activity was assessed by the Simple Clinical Colitis Activity Index for ulcerative colitis (UC) and Harvey Bradshaw Index for Crohn’s disease (CD). Multiple linear regression analysis was applied to examine associations between self-esteem and sociodemographic, clinical, and psychological factors.

**Results:**

In total, 411 of 452 (91%) patients had evaluable data and were included in this study. The mean scores on self-esteem, self-efficacy, total fatigue, anxiety, and depression were similar between UC patients and CD patients. Male gender, being employed, and higher self-efficacy were independently associated with higher self-esteem, whereas anxiety and depression were independently associated with lower self-esteem. Neither disease activity nor fatigue were associated with self-esteem in the final multiple regression analyses.

**Conclusion:**

Patient-centered interventions that improve self-esteem and reduce anxiety and depression seem to be important to optimize IBD management.

## Introduction

Chronic disease and its consequences may be a threat to personal and social identity and, in particular, a person’s self-esteem. Inflammatory bowel disease (IBD), which includes ulcerative colitis (UC) and Crohn’s disease (CD), is characterized by chronic inflammation of the gastrointestinal tract and results in recurrent symptoms such as abdominal pain and diarrhea, often with blood and mucus. In addition, patients may have systemic symptoms such as lack of appetite, weight loss, and fatigue, and a high proportion also report psychological comorbidities such as anxiety and depression [1–3]. IBD and its complications may have severe consequences for those afflicted, including frequent hospitalizations, surgery, sick leave, and an increased risk of work disability [4]. Disease onset is usually in adolescence/early-adulthood (15–35 years). Living with IBD may consequently be demanding and stressful, and it has been well demonstrated that IBD may negatively affect patients’ health-related quality of life (HRQoL) [5, 6].

For patients with IBD, symptoms may be experienced as socially embarrassing and as a stressor, which in turn has the potential to shape patients’ emotions, beliefs, and behaviors [7]. Indeed, it has been reported that IBD patients perceive that others hold stigmatizing views toward them and the disease, i.e., they feel different from others, shame, and discredited. Moreover, IBD stigma is associated with poorer health outcomes and exacerbates lower self-esteem and feelings of depression or anxiety [8].

Self-esteem is a self-concept that refers to the positive and negative feelings and evaluations we have about ourselves and represents our sense of self-worth [9]. While self-esteem may be threatened by the experience of various disease-related consequences, high self-esteem can be an important prerequisite for coping [10]. Previous research has shown that higher self-esteem is associated with a more positive affect in persons with chronic illness [11], and it buffers against depression, stress, and other negative emotions [11–13]. In adult IBD populations, studies on self-esteem are scarce, but stigma and body image dissatisfaction have been associated with lower self-esteem [8, 14–16]. In adolescents with IBD, self-esteem has been evaluated in several studies, and a meta-analysis revealed no significant differences in self-esteem between patients with IBD, healthy controls, and patients with other chronic diseases [13]. However, a more severe disease course has been found to be associated with lower self-esteem among adolescent IBD patients [17]. Previous studies have used self-esteem largely as a predictor variable and not as an outcome variable. We have not been able to find studies exploring factors associated with self-esteem in adult IBD populations.

Thus, the aim of this study was to explore self-esteem in an adult IBD population and to investigate associations between self-esteem and sociodemographic, clinical, and psychological factors.

## Methods

### Study design and population

In this cross-sectional study, participants were recruited from nine hospitals in the southeastern and western regions of Norway as part of an observational, multicenter study. The inclusion period lasted from March 2013 to April 2014. Inclusion criteria were age > 17 years; a verified diagnosis of IBD based on endoscopic, laboratory, and histological findings according to the Lennard–Jones criteria [18]; and the ability to read and understand Norwegian and to give written informed consent.

### Data collection

#### Sociodemographic and clinical data

Sociodemographic variables were self-reported by patients and included age, gender, marital status, educational level, work status, and smoking status. Clinical data were collected by interview, from medical records and from laboratory tests. The disease phenotype was classified according to the Montreal classification [19]. Disease activity was assessed through two disease activity indices: the Simple Clinical Colitis Activity Index (SCCAI) for UC [20] and the Harvey Bradshaw Index (HBI) for CD [21].

### Patient-reported outcomes

#### Rosenberg self-esteem scale

The Rosenberg Self-Esteem Scale (RSES) [22] assesses the patients’ global self-esteem and addresses overall feelings of self-worth and acceptance [9]. The RSES consists of ten statements with responses ranked from 1 ‘strongly agree’ to 4 ‘strongly disagree.’ The total score is the sum of item responses and ranges from 10 to 40, with higher scores representing higher self-esteem. The RSES has been translated into Norwegian and validated [23].

#### The fatigue questionnaire

The Fatigue Questionnaire (FQ) was developed by Chalder et al. [24] and has been translated into Norwegian and validated [25]. The FQ consists of 11 items. Responses are based on four options (0 = better than usual, 1 = no more than usual, 2 = worse than usual, and 3 = much worse than usual), and the dichotomized item scores were 0 for item response values 0 or 1 and 1 for item response values 1 or 2. Higher scores indicate higher levels of fatigue [24, 25].

#### The general self-efficacy scale

The General Self-Efficacy Scale (GSE) [26] measures a person’s optimistic self-beliefs in coping with the demands of life. The GSE consists of 10 statements that the respondents rate from 1 “completely agree” to 4 “completely disagree.” The GSE total score is calculated by summing each individual score (range 10 to 40). A higher score indicates stronger self-efficacy. The GSE has high reliability and validity [27].

#### Hospital anxiety and depression scale

The Hospital Anxiety and Depression Scale (HADS) was developed to assess anxiety and depression among diverse clinical and nonclinical hospital populations [28]. The HADS contains 14 questions, each of which is scored on a Likert scale from 0 to 3. The HADS subscales for anxiety (HADS-A) and depression (HADS-D) are scored between 0 and 21. The HADS has been translated into Norwegian, and this version has been validated [29].

### Statistical analysis

Differences between groups were assessed with the chi-square (*χ*^*2*^) test for categorical data and the independent sample *t* test for continuous variables with normal distributions. When the continuous variables had skewed distributions, they were described with medians and ranges, and differences between groups were tested using the nonparametric Mann–Whitney U test. Multiple linear regression analyses were used to determine the associations between self-esteem (dependent variable) and relevant sociodemographic, clinical, and psychological (independent) variables. In addition to gender and age, variables with *p* values < 0.15 in the univariate analyses were entered into a multiple linear regression model. None of the independent variables included in the final analyses were highly correlated (Pearson’s correlation coefficient > 0.7) with any other possible variable; thus, we assumed no multicollinearity. All tests were two-sided and analyzed using Statistical IBM SPSS Statistics for Windows version 25.0 (IBM Corp., Armonk, NY, released in 2017).

## Results

### Study sample

Of the 452 patients invited to participate in the study, 414 patients (91.6%) gave written informed consent. Three patients were excluded due to missing data, bringing the number included in the analyses to 411 (91%). No significant differences in gender or age were found between the patients excluded due to missing data and those included in the analyses (data not shown).

Patient characteristics are presented in Table 1. There were no significant differences between UC and CD patients with regard to age, gender, education level, or civil status. However, compared to UC patients, a higher proportion of CD patients were unemployed, had longer disease duration, and a higher proportion had undergone gastrointestinal surgery, including 15 patients with an ileostomy. The mean self-esteem, self-efficacy, total fatigue, anxiety, and depression scores were comparable between the two groups.

**Table 1:** Characteristics of IBD patients

Characteristics	IBD (*n* = 411)	UC (*n* = 180)	CD (*n* = 231)	*p* value
Sociodemographic characteristics				
Age (Mean, SD)	40.72 (12.77)	40.75 (12.57)	40.70 (12.96)	0.97
Gender *n* (%)				
Female	202 (49)	87 (48)	115 (50)	0.77
Male	209 (51			
Education				
University degree *n*, (%)	175 (43)	80 (45)	95 (41)	0.44
High school	234 (57)	98 (55)	136 (59)	
Civil status *n* (%)				
Married	322 (78.2)	146 (81)	176 (76)	0.19
Divorced, unmarried, widow(er)	88 (22)	33 (18)	55 (24)	
Work status *n* (%)				
Working/student	277 (68)	131 (73)	146 (63)	0.33
Not working	133 (32)	48 (27)	85 (37)	
Clinical characteristics				
HBI (Median, range)			4 (0 to 24)	
SCCAI (Median, range)		3 (0 to 17)		
Disease duration				
Median (range)	9 (0 to 50)	6 (0 to 46)	11 (0 to 50)	< 0.001
Location *n* (%)				
L1 terminal ileum			75 (32.5)	
L2 colonic			48 (20.8)	
L3 ileocolonic			76 (32.9)	
L4 upper tract only or modifier†			32	
Behavior *n* (%)				
B1 inflammatory			118 (51)	
B2 stricturing			83 (36)	
B3 penetrating			30 (13)	
Perianal† (yes)			51	
Extent *n* (%)				
E1 proctitis		20 (11.1)		
E2 left-sided		58 (32.2)		
E3 extensive		102 (56.7		
On medication *n* (%)				
Biological	167 (40)	53 (29)	114 (49)	
Immunomodulator	136 (33)	47 (26)	89 (39)	
Gastrointestinal surgery *n* (%)				
Yes		10 (6)	108 (47)	
No		170 (94)	123 (53)	< 0.01
Ileostomy	15 (3.6)	2 (1)	13 (5.6)	
Psychological factors				
Self-esteem (Mean, SD)	30.39 (4.7)	30.58 (4.5)	30.24 (4.9)	
Self-efficacy (Mean, SD)	29.35 (5.8)	29.23 (5.7)	29.43 (5.8)	
HADS–Anxiety (Mean, SD)	6.04 (3.7)	6.29 (3.7)	5.84 (3.7)	
HADS–Depression (Mean, SD)	3.79 (3.8)	3.70 (3.3)	3.86 (3.3)	
Total fatigue score (Mean, SD)	15.9 (5.4)	16.1 (5.2)	15.8 (5.6)	

*IBD* inflammatory bowel disease; *UC* ulcerative colitis; *CD* Crohn's disease; *HBI* Harvey Bradshaw Index; *SCCAI* Simple Clinical Colitis Activity Index. †Upper tract modifier and perianal disease coexist with other location categories

Continuous variables were estimated by student’s *t* test and the Mann–Whitney *U* test, and the Chi-squared test (*X*^2^) was used to compare proportions. *p* value estimated between CD and UC patients

### Univariate and multiple associations with self-esteem

The univariate and multiple associations with self-esteem are shown in Table 2 for UC patients and Table 3 for CD patients. In both disease groups, increased self-esteem was associated with male gender, being employed and higher self-efficacy. Furthermore, lower self-esteem was associated with anxiety, depression, total fatigue, and disease activity. In addition, lower self-esteem was associated with gastrointestinal surgery in UC patients, and higher self-esteem was associated with being in a relationship and with a higher educational level in CD patients.

**Table 2:** Univariate and multiple linear regression analyses with self-esteem as the dependent variable in patients with ulcerative colitis (*n* = 180)

Variable	Univariate		Multiple	
	*β* (95% CI)	*p value*	*β* (95% CI)	*p value*
Age	0.03 (-0.02,0.08)	0.25	0.02 (-0.02,0.07)	0.33
Gender	− 2.57 (− 3.86, 1.29)	< 0.001	− 1.45 (− 2.55, 0.34)	0.01
Education level	0.99 (− 0.36,2.35)	0.15	0.43 (− 0.63,1.50)	0.43
Civil status	− 0.66 (− 2.40,1.09)	0.46	−	−
Work status	− 2.43 (− 3.89, 0.97)	0.001	− 1.25 (− 2.52,0.01)	0.05
Disease activity	− 0.40 (− 0.61, − 0.20)	< 0.001	− 0.03 (− 0.23,0.17)	0.76
Gastrointestinal surgery	− 2.95 (− 5.80, 0.10)	0.03	− 1.29 (− 3.76,1.08)	0.29
Disease duration	0.00 (− 0.80,0.84)	0.96	−	−
Anxiety	− 0.60 (− 0.76, 0.45)	< 0.001	− 0.29 (− 0.47, 0.10)	0.003
Depression	− 0.60 (− 0.79, 0.42)	< 0.001	− 0.26 (− 0.47, 0.05)	0.01
Total fatigue	− 0.29 (− 0.41, 0.17)	< 0.001	− 0.01 (− 0.14, 0.05)	0.92
Self-efficacy	0.39 (0.29,0.49)	< 0.001	0.26 (0.16,0.35)	< 0.001

*β*, estimated beta coefficient = association with self-esteem while controlling for all other variables in the final regression model. In addition to gender and age, variables in the univariate analyses with *p* < 0.15 were included in the final regression model

*Coding* Gender, men = 0, women = 1; education level, <  = 12 years = 0, >  12 years = 1; civil status, married/in a relationship = 0, divorced/unmarried/widow(er) = 1; work status, working/student = 0, not working = 1; gastrointestinal surgery, no = 0, yes = 1

**Table 3:** Univariate and multiple linear regression analyses with self-esteem as the dependent variable in patients with Crohn’s disease (*n* = 231)

Variable	Univariate		Multiple	
	*β* (95% CI)	*p value*	*β* (95% CI)	*p value*
Age	0.03 (− 0.02,0.08)	0.27	0.006 (− 0.03,0.04)	0.76
Gender	− 2.55 (− 3.81, 1.29)	< 0.001	− 1.87 (− 2.86, 0.88)	< 0.001
Education level	1.38 (0.06,2.69)	0.04	0.11 (− 0.82,1.04)	0.82
Civil status	− 1.66 (− 3.19, 0.12)	0.03	− 0.72 (− 1.81,0.38)	0.20
Work status	− 2.19 (− 3.52, 0.86)	0.001	0.19 (− 0.90,1.28)	0.73
Disease activity	− 0.36 (− 0.50, 0.17)	< 0.001	0.006 (− 0.13,0.14)	0.93
Gastrointestinal surgery	0.14 (− 1.45,1.17)	0.83	−	−
Disease duration	0.007 (− 0.06,0.07)	0.84	−	−
Anxiety	− 0.58 (− 0.73, 0.42)	< 0.001	− 0.16 (− 0.31, 0.01)	0.03
Depression	− 0.83 (− 1.00, 0.66)	< 0.001	− 0.51 (− 0.69, 0.34)	< 0.001
Total fatigue	− 0.31 (− 0 − 42, 0.21)	< 0.001	− 0.05 (− 0.15,0.05)	0.31
Self- efficacy	0.48 (0.39,0.57)	< 0.001	0.37 (0.28,0.45)	< 0.001

*β,* estimated beta coefficient = association with self-esteem while controlling for all other variables in the final regression model. In addition to gender and age, variables in the univariate analyses with *p* < 0.15 were included in the final regression model

*Coding* Gender, men = 0, women = 1; education level, <  = 12 years = 0, > 12 years = 1; civil status, married/in a relationship = 0, divorced/unmarried/widow(er) = 1; work status, working/student = 0, not working = 1; gastrointestinal surgery, no = 0, yes = 1

The multiple linear regression analyses showed, in both UC and CD patients, that higher self-efficacy scores and male gender were independently associated with higher self-esteem. In UC patients, being employed was additionally independently associated with higher self-esteem. Increased anxiety and depression scores were independently associated with lower self-esteem. Clinical factors and fatigue were not significantly associated with self-esteem in the final regression analyses.

## Discussion

The key findings in this study were that male gender, being employed, and higher self-efficacy were associated with higher self-esteem, whereas anxiety and depression were associated with lower self-esteem. Clinical factors were not associated with self-esteem.

The mean self-esteem score found among the IBD patients in our study (30, range 15–40) is comparable to previously reported findings in an adult IBD population (mean self-esteem score of 33; range 27–34) [30] and to patients with irritable bowel syndrome (mean self-esteem score 30; range 24–36) [30] and multiple sclerosis (mean self-esteem score 29; range 16–40) [31]. This may suggest that some factors contributing to the level of self-esteem are common across diseases, such as anxiety, depression, and self-efficacy.

The strong association between self-efficacy and self-esteem found in this study is in line with self-concept theories [32] and results from earlier research in a variety of chronic disease populations [31, 33, 34]. The direction of the associations found in our study cannot be evaluated due to the cross-sectional study design. However, self-efficacy influences people’s choices and behavior [35]; thus, it may be hypothesized that self-esteem impacts a person's belief in their ability to accomplish a specific goal or task (self-efficacy) [31]. In this way, self-esteem may be threatened by experiencing illness-related consequences or by decreased coping with everyday life challenges. To account for the variability in disease-related demands and illness-related concerns between different chronic diseases, an IBD-specific self-efficacy scale has been developed and validated for patients in both adult and adolescent populations [36–38]. The scale relates to the unique self-management requirements of IBD patients, such as managing stress and emotions, managing medical care, managing symptoms and disease, and maintaining remission [36]. The strong relationship between self-esteem and self-efficacy found in our study indicates that they are both important constructs in disease management. When developing targeted interventions, it may be important to include both self-efficacy and self-esteem to identify areas where IBD patients may need more support for self-management, such as in transitioning from pediatric to adult care, developing emotional coping skills, and navigating medical care. Self-esteem and self-efficacy are also positively associated with other personal characteristics, such as sense of coherence, internal locus of control, achievement motivation, self-control, optimism, satisfaction, and emotional stability [35, 39].

The strong negative relationship between self-esteem and depression and anxiety found in our study is consequently not surprising and aligns with prior studies. In a meta-analysis including 77 longitudinal studies, low self-esteem predicted symptoms of depression and anxiety across gender, age, and populations [40]. The strongest relationship was found between low self-esteem and depression [41]. Several theoretical frameworks explain this relationship, suggesting that self-esteem has an important role in a person’s positive and negative affective state and that low self-esteem is a vulnerability factor for the development of depression [9, 40, 42]. This is important for patients with IBD because large cohort studies have shown that their rates of depression are significantly higher compared to healthy populations [43]. Furthermore, a systematic review revealed that both anxiety and depression are associated with clinical factors such as a more severe disease course, increased disease activity, more frequent disease flares, and increased hospitalization rates, [3, 43], all of which cause disease unpredictability and impact patients’ daily functioning and HRQoL. This shows that there is a need to strengthen disease management by including evidence-based individualized psychosocial support in routine follow-up and care [44].

Fatigue was associated with lower self-esteem in the univariate analysis. Increased fatigue scores have been consistently related to depressive symptoms in previous IBD studies (45–46). As self-esteem was strongly associated with depression in the current study, one might expect a relationship between increased fatigue and lower self-esteem as well. However, in the final regression analyses, neither total fatigue scores nor the mental and physical fatigue subscores were independently associated with self-esteem [45, 46]. To our knowledge, our study is the first to evaluate the association between self-esteem and fatigue in an IBD population. The ability to controlling for interactions between fatigue and depression in the multiple regression analyses may have elucidated the effect of fatigue on self-esteem.

Male gender and being employed were factors independently associated with higher self-esteem in our study. The fact that males reported higher levels of self-esteem than females is consistent with previous research [23, 47]. Employment is associated with feelings of worthiness and societal usefulness, income, and autonomy, and is thus an important element in a person’s identity. Education level and being in a relationship were associated with higher self-esteem in the univariate analysis but not in the final regression models. This finding is in contrast to results from a study of the general population in Norway, which found lower socioeconomic status, not having a cohabiting partner, unemployment, and disability were each uniquely associated with lower levels of self-esteem [41]. However, the direction of the associations was unclear. It may be that high self-esteem influences socioeconomic status.

Clinical factors (disease activity, gastrointestinal surgery, having an ileostomy) did not independently influence self-esteem in our study. This is in line with a previous study of IBD patients [30]. The impact of disease symptoms and physical consequences of IBD have been shown in earlier studies to affect psychological and social well-being [48]. Patients with higher disease activity reported lower self-efficacy and increased psychological distress in a study of IBD patients using the disease-specific self-efficacy scale in two different samples of IBD patients: one online sample recruited through websites and one outpatient sample. Thus, it could be hypothesized that disease activity also affects the level of self-esteem. Having ongoing disease symptoms such as diarrhea, bowel incontinence, and urge can be embarrassing and can affect someone’s interest in socializing. Lower self-esteem predicted more symptom interference and reported activity restrictions in a study of patients with rheumatoid arthritis and asthma [11]. However, we did not ask the patients about symptom burden or whether the symptoms interfered with daily functioning or well-being. Furthermore, in our study, nearly one-third of the IBD patients had undergone gastrointestinal surgery, and 15 patients had an ileostomy. In a study by Taft et al. [16], patients reported that disease symptoms such as diarrhea, bowel sounds, bowel incontinence, and urge were embarrassing and that gastrointestinal surgery affected the patient’s body image. However, there were no significant differences in self-esteem scores between the IBD patients with and without ileostomy. We did not assess body image concerns in our study, which could add important information to explain these relationships.

The study has some limitations. The complex relationships between the factors examined in this study could not be well explored using a cross-sectional sign. It may be that self-esteem varies over time depending on social, psychological, and clinical status. A longitudinal design may provide a better understanding of how these different factors relate to each other. Moreover, we did not assess body image concerns, which could add important information to the findings in our study.

## Conclusion

In this adult IBD population, self-esteem was strongly associated with male gender, anxiety, depression, and self-efficacy but not with clinical factors. Patients with IBD vary by disease type, disease severity, surgical history, and sociodemographic and psychological presentation. Therefore, the care and follow-up of patients need to be individualized. Regardless of diagnosis, psychological factors were the strongest contributors to the variance in self-esteem in our study. Patient-centered interventions that improve self-esteem and reduce anxiety and depression seem to be important to optimize IBD management.
